# Ultrastructure of the fertilized egg envelopes in *Hemigrammus erythrozonus*, Characidae, Teleostei

**DOI:** 10.1186/s42649-019-0010-8

**Published:** 2019-08-20

**Authors:** Byung Soo Chang, Eun-Kyung Choi, Hyun-Wook Kim, Dong Heui Kim

**Affiliations:** 1 Department of Cosmetology, Hanseo University, Seosan, 31962 Korea; 2 College of Life Sciences and Biotechnology, Korea University, Seoul, 02841 Korea; 3 Department of Pathology, Seoul National University, Seoul, 03080 Korea; 4 Medical Science Research Center, Korea University College of Medicine, Seoul, 02841 Korea; 5 Department of Environmental Medical Biology, Yonsei University Wonju College of Medicine, Wonju, 26426 Korea

**Keywords:** Characidae, Egg envelope, Fertilized egg, *Hemigrammus erythrozonus*, Ultrastructure

## Abstract

We examined the morphology and ultrastructures of fertilized egg envelopes of glowlight tetra (*Hemigrammus erythrozonus*) belong to Characidae using light and electron microscopes.

The fertilized eggs were spherical, transparent, demersal, adhesive, and have no oil droplet. The perivitelline space was well-developed and the micropyle was surrounded by 15–20 uplifted lines of egg envelope in a spoke like pattern. The outer surface of egg envelope was rough side with grooves. Also, the total thickness of the fertilized egg envelope was about 2.1–2.3 μm, and the fertilized egg envelope consisted of two layers, an outer adhesive electron-dense layer with grooves and three feather-like lamellae layers. Collectively, these morphological characteristics of fertilized egg and micropyle with spoke-like structure showed family Characidae specificity, and ultrastructures of outer surface and section of fertilized egg envelope showed species specificity.

## Introduction

The glowlight tetra (*Hemigrammus erythrozonus* Durbin 1909) is a teleost belong to Characidae, Characiformes, and Actinopterygii. And this species is distributed on Essequibo River, Guyana, South America (Wikipedia contributors 2019). The oogenesis of *Hemigrammus erythrozonus* have similar characteristics in increase in cell size, the formation and accumulation of yolk, and the decrease of basophilia a in the cytoplasm such as other teleost (Lee et al. 2008). In teleost, the morphology of fertilized eggs is known to differ according to the family (Joo and Kim 2013; Choi et al. 2019). The ultrastructures of outer surface and section from fertilized egg envelope show species, genus or family specificity (Kim et al. 1998, 1999; Joo and Kim 2013; Kwon et al. 2015; Choi et al. 2019).

In Characidae, the ultrastructure of fertilized egg envelope has been studied in *Hemigrammus ocellifer*, *Gymnocorymbus ternetzi, Hemigrammus caudovittatus* (Kim et al. 1996) and *Hyphessobrycon serpae* (Kim et al. 2005). In these four species belonging to Characidae, the fertilized eggs are transparent, demersal, and spherical type. The fertilized eggs of *Hemigrammus ocellifer*, *Gymnocorymbus ternetzi*, *Hyphessobrycon serape* are adhesive type, but that of *H. caudovittatus* is non-adhesive type. Also, ultrastructures of outer surface and section of fertilized egg envelope are different according to the species each other although the species are belong to same genus. In general, teleost egg has a micropyle on the animal pole, it plays a role of canal of sperm without acrosome for fertilization (Yanagimachi et al. 2017). In our previous research, we found special micropyle structure from fertilized egg envelope of 4 species all belonging to Characidae. The micropyle was surrounded by protruded egg envelopes in a radiated form such as spoke (Kim et al. 1996, 2005).

*Hemigrammus erythrozonus* was studied on visual antipredator alarm signal (Brown et al. 1999) and oogenesis (Lee et al. 2008). There is no study on the ultrastructure of fertilized egg envelope because it is hard to get fertilized eggs from this species. Also, it is hard for sure to these morphological characteristics and micropyle with spoke-like structure show family specificity because of very few research samples. So, we studied the morphology of fertilized egg, and compared the ultrastructures of outer surface, micropyle, and section of fertilized egg envelopes under the light and electron microscopes from the other a species, *Hemigrammus erythrozonus*, Characidae to find out whether these structures have the species, genus or family specificity.

## Materials and methods

### Animals

The glowlight tetra, *Hemigrammus erythrozonus* (*n* = 30, total length: 3.5–4.0 cm) used in this study were purchased from SanHo Aquarium (Wonju, Korea). The tap water used for rearing was treated with Fritz-guard (Fritz Co. Ltd., USA) to remove chlorine, and its temperature and pH were maintained at 25 ± 0.5 °C and 7.0 ± 0.5, respectively. Biological filtration was performed using a sponge filter (LS-M, Premium sponge filter™, Leglass, Korea), and excrement settled to the bottom of the water tank was eliminated by exchanging one-quarter of the water each day. An artificial light was illuminated for ten hours per day to simulate a daytime environment, and live tubifex and Brine Shrimp Plus Flakes™ (Ocean Nutrition, U.S.A.) were provided as food two times per day, at 9 a.m. and 5 p.m.

### Collection of fertilized eggs

The water of breeding tank was made by mixing rearing water and purified water by reverse osmosis, then was treated with peat moss for cultivation. It was adjusted to 24 ± 0.5 °C, 30 ppm, and pH 5.0 ± 0.5, respectively. One day prior to the collection of fertilized eggs, male and female of experimental fishes in a 2 to 1 ratio were put into a glass water tank (45X30X30cm) with a net. The fertilized egg was corrected at the bottom of glass water tank after spawning. Fertilized eggs which confirmed the formation of perivitelline space were measured for size (*n* = 20) under digital microscope (AD-7013MZT, Dino-Lite, Anmo, Taiwan) and used in this study as experimental samples.

### Electron microscopy

For transmission electron microscope (TEM) observation, fertilized eggs were fixed in 2.5% glutaraldehyde in 0.1 M phosphate buffer (pH 7.4) for 2 h at 4 °C. After prefixation, the specimens were washed twice in the same buffer solution for 20 min. and then postfixed in 1% osmium tetroxide solution in 0.1 M phosphate buffer solution (pH 7.4) for 2 h at room temperature. Specimens were dehydrated in ethanol, cleared in propylene oxide, and embedded in an Epon mixture. Ultrathin sections of embedded fertilized egg envelope were taken with an ultramicrotome (Ultracut E, Reichert-Jung, Austria) at a thickness of about 60 nm. The ultrathin sections were mounted onto copper grids, double stained with uranyl acetate followed by lead citrate, and observed with a transmission electron microscope (JEM-1400, JEOL, Japan).

For scanning electron microscope observation, prefixation, postfixation and dehydration were conducted by following the same procedure as that for TEM. The samples were replaced with tert-butyl alcohol and freeze dried (ES-2030, Hitachi, Japan). The samples were coated with Pt by ion sputter (E-1045, Hitachi, Japan). Subsequently, the fertilized eggs were observed under the scanning electron microscope (S-4700, Hitachi, Japan).

## Results and discussion

### Morphology of fertilized eggs

**Fig. 1:**
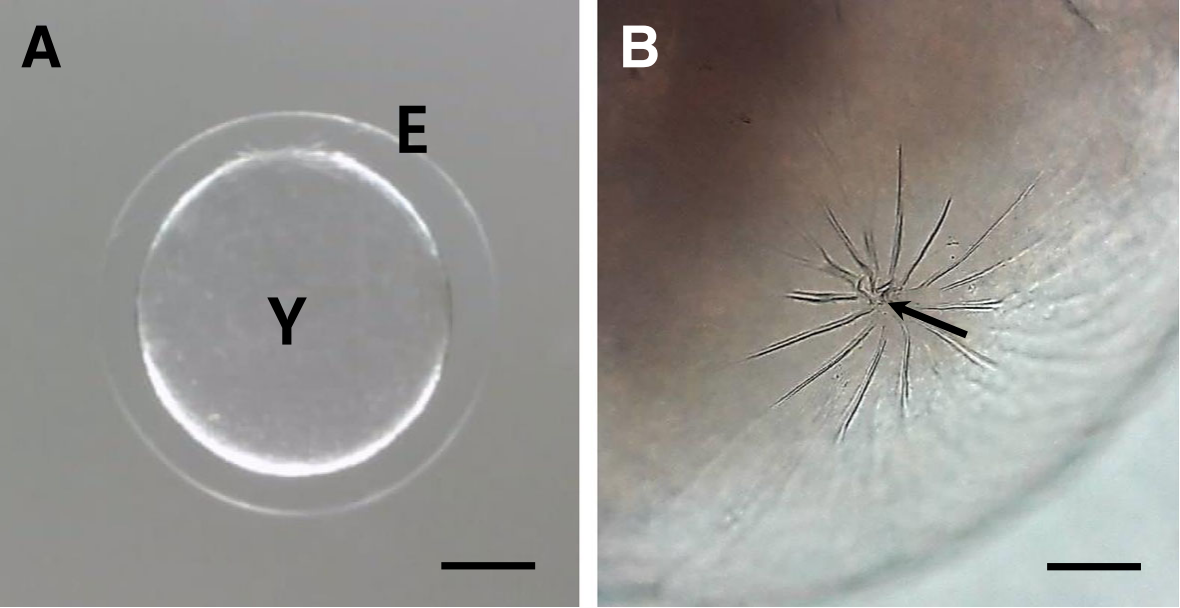
The fertilized eggs (**a**) and micropyle (**b**) of glowlight tetra, *Hemigrammus erythrozonus*. The micropyle was surrounded by spoke-like structure. E; egg envelope, Y; yolk, an arrow; micropyle (Scale bar = A; 0.3 mm, B; 0.1 mm)

The fertilized eggs of *Hemigrammus erythrozonus* were spherical, transparent, demersal, adhesive, and have no oil droplet. The size of fertilized egg was 1.11 ± 0.02 mm (*n* = 20). At low magnification, the outer surface of fertilized egg envelope perivitelline space was well-developed (Fig. 1a). Also, a micropyle was located in the area of animal pole. The micropyle (arrow in Fig. 1b) was surrounded by spoke like structure. The fertilized eggs of fishes belong to Characidae and Cyprinidae have similar morphology although the size of fertilized eggs is different (Kim et al. 1996, 2005; Joo and Kim 2013). The most of fertilized eggs from Characidae are spherical, demersal, adhesive, and transparent. But that of *H. caudovittatus* is non-adhesive type (Kim et al. 1996, 2005). In teleost, the morphology of fertilized eggs is same according to the family (Joo and Kim 2013; Choi et al. 2019) or genus (Kwon et al. 2015, 2017; Choi et al. 2019).

### Structure of micropyle

**Fig. 2:**
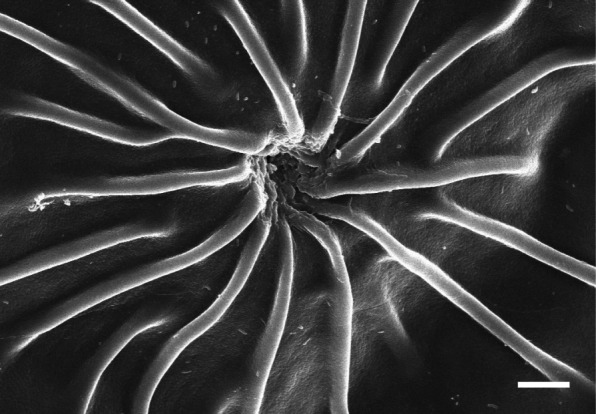
Scanning electron micrographs of a micropyle on the area of the animal pole from fertilized egg of *Hemigrammus erythrozonus* (Scale bar = 10 μm)

In most teleost, a micropyle is located on the animal pole of fertilized egg. We found special structure surrounding micropyle on fertilized eggs of *Hemigrammus ocellifer*, *Gymnocorymbus ternetzi, Hemigrammus caudovittatus* (Kim et al. 1996) and *Hyphessobrycon serpae* (Kim et al. 2005) belong to Characidae in our previous research. In this study, a micropyle was surrounded by 15–20 uplifted lines of egg envelope in a spoke like pattern with narrow ends toward center under scanning electron microscope (Fig. 2). The inter-layer spacing of this uplifted line of egg envelope is known to larger in lower side than the upper side, and center of ridge base layer was filled with low-electron density material (Kim et al. 1996). Therefore we suggest that this micropyle structure shows family Characidae specificity because of micropyle of *Hemigrammus erythrozonus* belong to same Characidae have identical structure with those of other species belong to same Characidae. The micropyle of *Zacco platypus*, Cyprinidae is surrounded by five peaks of hill structures (Deung et al. 2000). But there are no special structures around the micropyle in most teleost. The micropyles are funnel shape in Belontiidae (Kim et al. 1999) and a plate coral mouth shape in genus *Nothobranchius* (Kwon et al. 2015) but, morphology of micropyle is differ according to the species in Cyprinidae. Therefore, structure of micropyle seems to be family specificity, genus specificity or species specificity.

### Outer surfaces of the fertilized egg envelopes

**Fig. 3:**
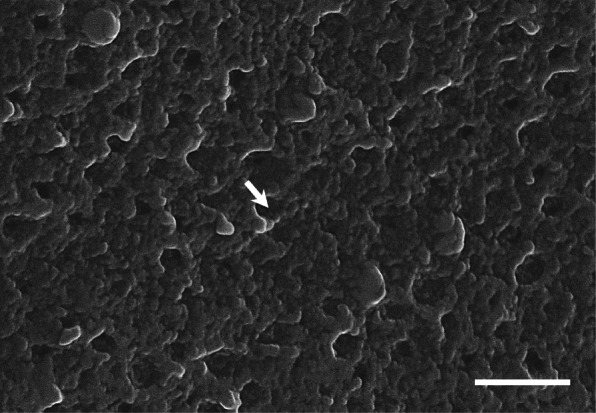
Scanning electron micrographs of outer surface on the egg envelopes of *H. erythrozonus.* Grooves (arrow) were distributed on the outer surface (Scale bar = 1 μm)

At the low magnification, the adhesive outer surface of fertilized egg envelope was smooth, but outer surface was rough side with grooves about 0.3–0.4 μm in diameter at higher magnification and the grooves were distributed in 4–5 per 1 μm^2^ (Fig. 3). This fine structures of adhesive outer surface from *Hemigrammus erythrozonus* are very similar with those of *Gymnocorymbus ternetzi* and *Hyphessobrycon serape,* but have different structure from those of *Hemigrammus ocellifer* and *H. caudovittatus* belong to Characidae (Kim et al. 1996, 2005). This result also demonstrate that outer surface structures can be same even if the species belongs to different genus. The outer surface of egg envelope from species belong to genus *Trichogaster, Nothobranchius* and *Corydoras* have same structure (Kwon et al. 2015, 2017; Choi et al. 2019). Therefore these ultrastructures showed genus specificity. Also, differences of ultrastructure and the number of structures per unit area between *Danio rerio* and *Dnaio rerio* var*. frankei* belong to Cyprinidae could be differentiated by species variation (Joo and Kim 2013).

### The section of fertilized egg envelope

**Fig. 4:**
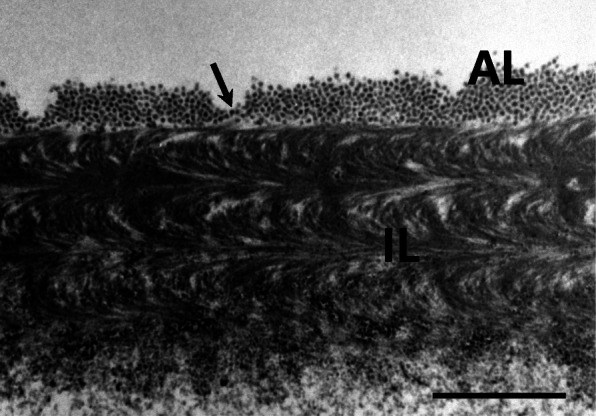
Transmission electron micrographs of the fertilized egg envelopes from *H. erythrozonus*. The fertilized egg envelope consisted of two layers, an outer adhesive electron-dense layer (AL) with grooves (arrow) and an inner three feather-like layers (IL) (Scale bar = 1 μm)

The total thickness of the fertilized egg envelope was about 2.1–2.3 μm, and the fertilized egg envelope consisted of two layers, an outer adhesive electron-dense layer with grooves and an inner three feather-like lamellae layers. The outer surface of egg envelope looked like a sand layer with grooves in scanning electron microscopic observation and the thickness of outer layer was about 0.28–0.31 μm (Fig. 4). In other species belong to Characidae, the fertilized egg envelopes of *Hemigrammus ocellifer*, *H. caudovittatus*, and *Hyphessobrycon serpae* consisted of 3 layers, and that of *Gymnocorymbus ternetzi* consisted of 2 layers. But, the number of the inner layer of egg envelope is different according to the species. The number of inner layer of *Hemigrammus ocellifer* was three, that of *Gymnocorymbus ternetzi* was four, that of *H. caudovittatus* was five, and inner layer of *Hyphessobrycon serpae* consisted of 5–6 layers (Kim et al. 1996, 2005). In this study, morphological characteristics of this outer surface and total number of egg envelope are same with that of *Gymnocorymbus ternetzi* belong to different genus but, the number and structure of inner layer are different. We suggest that the ultrastructure of section of fertilized egg envelope can be seem to be shows species specificity in Characidae, because of the fine structures of section of fertilized egg envelope are different in all species. But five species including the previous research species are very limeted, so more research is needed for more information in Characidae.

The sections of egg envelope may have same fine structures in family, Belontiidae (Kim et al. 1999), Nothobranchiidae (Kwon et al. 2015, 2017), and Callichthyidae (Choi et al. 2019). The fertilized egg envelopes of these 4 families consisted of 2 layer, but the structure of inner layer are different according to the family. The inner layer in Belontiidae is single serrated layer, those of Cichlidae, Nothobranchiidae and Callichthyidae are multi-layer. Also, the outer envelope structures are showed family specificity, because the outer envelopes in Cichlidae, Nothobranchiidae and Callichthyidae have different structure. That of Cichlidae is adhesive reticular structures, that of Nothobranchiidae is adhesive filament, and that of Calichthyidae is adhesive protuberances. In general, feather-like and multilayer structures can be easily seen in many ultrastructural research on fertilized egg envelope, but are not understood for accurate 3D structure. Therefore, more research is needed using a 3D tomography technique.

Collectively, these morphological characteristics of fertilized egg and micropyle with spoke-like structure showed family Characidae specificity including results before (Kim et al. 1996, 2005), and ultrastructures of outer surface and section of fertilized egg envelope showed species specificity.

## Conclusions

The external shapes of fertilized egg and ultrastructures of micropyle, surrounded by uplifted lines of egg envelope in a spoke-like pattern were same structures including our previous research on Characidae. In conclusion, these morphological characteristics of fertilized egg and micropyle with spoke-like structure showed family Characidae specificity, and ultrastructures of outer surface and section of fertilized egg envelope showed species specificity.

## Data Availability

No applicable.
